# MEX3A contributes to development and progression of glioma through regulating cell proliferation and cell migration and targeting CCL2

**DOI:** 10.1038/s41419-020-03307-x

**Published:** 2021-01-04

**Authors:** Chao Yang, Haoqiang Zhan, Yiqing Zhao, Yasong Wu, Lisha Li, Heping Wang

**Affiliations:** 1 Department of Neurosurgery, First Affiliated Hospital of Sun Yat-Sen University, Guangzhou, 510080 China; 2 Department of Neurosurgery, The Six Affiliated Hospital of Sun Yat-Sen University, Guangzhou, China; 3 Department of Neurosurgery, TongJi hospital of TongJi Medical College, Huazhong University of Science and Technology, Hankou, Wuhan 430030 China; 4 The Key Laboratory of Pathobiology, Ministry of Education, College of Basic Medical Sciences, Jilin University, Changchun, 130012 China

**Keywords:** Cell biology, Medical research

## Abstract

Glioma is one of the most commonly diagnosed intracranial malignant tumors with extremely high morbidity and mortality, whose treatment was seriously limited because of the unclear molecular mechanism. In this study, in order to identify a novel therapeutic target for glioma treatment, we explored the functions and mechanism of MEX3A in regulating glioma. The immunohistochemical staining of MEX3A in glioma and normal tissues revealed the upregulation of MEX3A and further indicated the relationship between high MEX3A expression and higher malignancy as well as poorer prognosis of glioma. In vitro loss-of-function and gain-of-function experiments comprehensively demonstrated that MEX3A may promote glioma development through regulating cell proliferation, cell apoptosis, cell cycle, and cell migration. In vivo experiments also suggested the inhibition of glioma growth by MEX3A knockdown. Moreover, our mechanistic study identifies CCL2 as a potential downstream target of MEX3A, which possesses similar regulatory effects on glioma development with MEX3A and could attenuate the promotion of glioma induced by MEX3A overexpression. Overall, MEX3A was identified as a potential tumor promoter in glioma development and therapeutic target in glioma treatment.

## Introduction

Glioma, originating from brain glial cells, is the most common malignant tumor in the central nervous system, seriously threatening human health^1^. The statistical data showed that the incidence rate of primary intracranial tumors is about 10–21/100,000, in which glioma accounts for 40–50%^2–5^. The World Health Organization classifies gliomas into grades I–IV, among which grades I and II are low-grade gliomas, grades III and IV are high-grade gliomas, and grade IV glioma was also known as glioblastoma multiforme (GBM)^6^. At present, surgical resection, in combination with postoperative radiotherapy and adjuvant chemotherapy, is the standard treatment strategy for patients with high-grade glioma. However, due to the strong proliferative activity and high invasiveness of glioma, the therapeutic efficacy is very poor. The median survival period of GBM patients is only about 14 months, and the 5-year mortality rate is more than 95%^7^. The occurrence and development of glioma are multiple progressive processes. In the past few decades, fundamental research has revealed that gene mutation or amplification is widely involved in the occurrence and development of glioma. Preclinical studies showed that targeting the core signaling pathways of GBM, such as receptor tyrosine kinase, RAS, and PI3K, has gradually become a promising therapeutic strategy^8,9^. Therefore, the development of new key targets to promote the development of targeted therapy is of great significance for the treatment of glioma.

MEX3A is a member of the MEX3 family, which widely exists in mammalian cells and encodes four different genes, MEX3A, MEX3B, MEX3C, and MEX3D. It is reported that MEX3 was first discovered by Draper et al.^10^ in *Caenorhabditis elegans*, and then homologous proteins of MEX3 (MEX3A, MEX3B, MEX3C, and MEX3D) were also found in human and mice^11^. The MEX3 protein of mammals is composed of two RNA-binding KH domains and a ring domain with E3 ubiquitin ligase activity at the C terminal. The ring finger domain endows human MEX3 protein the ability to regulate ubiquitination of target proteins and then influencing their protein stability and subcellular localization^12–14^. These four MEX3 proteins are involved in many post-transcriptional regulations. Among them, MEX3C has been proved to be associated with many diseases, including hypertension and cancer^15,16^. Xue et al.^17^ reported that MEX3B is involved in the degradation of Run3 induced by lncRNA HOTAIR, and Run3 protein can effectively inhibit the migration and invasion of gastric cancer cells. Although the relationship between MEX3A and several types of malignant tumors has been revealed^18–20^, its association with glioma is still unknown.

In this study, we explored the clinical significance of MEX3A in glioma through immunohistochemistry (IHC) and statistical analysis. Based on the construction of MEX3A- knockdown cell models or xenografts, in vitro and in vivo loss-of-function studies were carried out to clarify the exact roles of MEX3A in the regulation of phenotypes of glioma. A gene chip microarray together with Ingenuity Pathway Analysis (IPA) analysis was performed to screen and establish the relationship between MEX3A and the potential downstream target CCL2. Moreover, the gain-of-function study of MEX3A and phenotype “rescue” experiments of the MEX3A/CCL2 axis were used for verifying the regulatory effects of MEX3A/CCL2 in glioma. Our results demonstrated that MEX3A may promote the development of glioma through regulating CCL2 expression.

## Results

### MEX3A is upregulated in glioma tissues and expressed in glioma cells

**Fig. 1:**
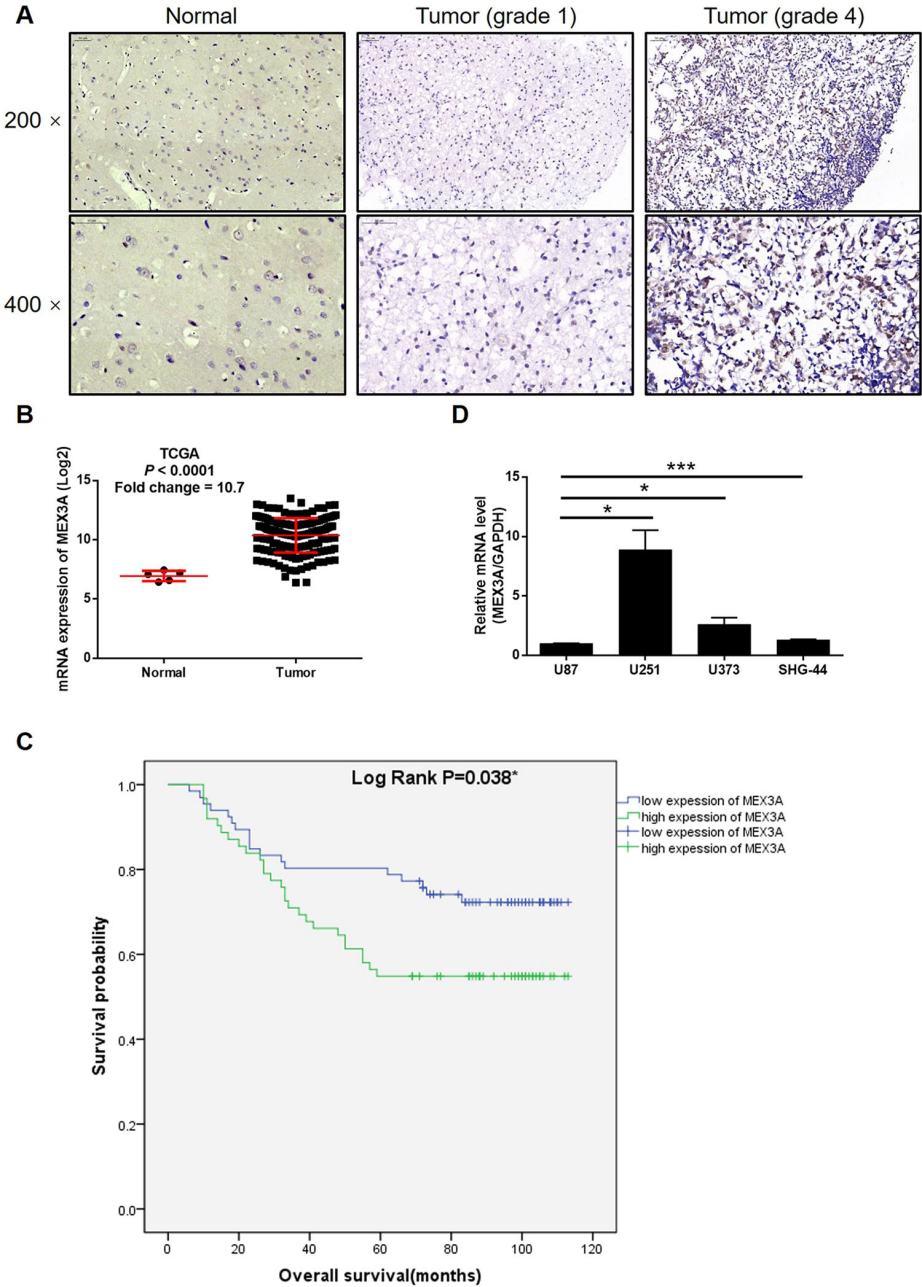
MEX3A was upregulated in glioma tissues and expressed in glioma cells. **A** The expression level of MEX3A was detected by IHC analysis in glioma and normal tissues. **B** The mining of RNA-seq data of TCGA showed the upregulated mRNA expression of MEX3A in tumor tissues of glioma patients compared with that in normal tissues. **C** The association between MEX3A expression and prognosis of glioma patients was evaluated with Kaplan–Meier survival analysis. **D** The mRNA expression of MEX3A in U87, U251, U373, and SHG-44 cell lines was detected by qPCR. The representative images were selected from at least three independent experiments. Data were shown as mean ± SD. *P < 0.05, **P < 0.01, ***P < 0.001.

First of all, in order to explore the role of MEX3A in glioma, the quantification of its expression in glioma and normal tissues was measured by IHC analysis. As shown in Fig. 1A and Table 1, it was clarified that glioma tissues expressed a higher level of MEX3A in comparison with normal tissues. Meanwhile, the expression profiling data collected from The Cancer Genome Atlas (TCGA) also demonstrated a 10.7-fold higher MEX3A expression in tumor tissues relative to normal tissues (*P* < 0.001, Fig. 1B). Moreover, the correlation analysis between MEX3A expression and clinical parameters of glioma patients revealed a positive correlation between higher MEX3A levels and higher tumor malignancy (Table 2 and Table S4). The Kaplan–Meier survival analysis, showing the poorer prognosis of patients with higher MEX3A expression, further suggested the involvement of MEX3A in glioma development (Fig. 1C). Finally, the endogenous expression of MEX3A in glioma cell lines, including U87, U251, U373, and SHG-44, was detected and proved (Fig. 1D). All these results implied that the role of MEX3A in glioma deserves further investigation.

**Table 1:** Expression patterns of MEX3A in glioma and normal tissues revealed in immunohistochemistry analysis.

MEX3A expression	Tumor tissue		Normal tissue	
	Cases	Percentage	Cases	Percentage
Low	66	51.6%	24	100%
High	62	48.4%	0	–

*P* < 0.001.

**Table 2:** Relationship between MEX3A expression and tumor characteristics in patients with glioma.

Features	No. of patients	MEX3A expression		*P* value
		low	high	
All patients	128	66	62	
*Age (years)*				0.424
<42	63	35	28	
≥42	64	31	33	
*Gender*				0.626
Male	84	42	42	
Female	44	24	20	
*Tumor recurrence*				0.102
No	57	34	23	
Yes	71	32	39	
*Grade*				0.027*
I	14	10	4	
II	59	33	26	
III	36	16	20	
IV	19	7	12	

### MEX3A knockdown inhibited glioma development in vitro

**Fig. 2:**
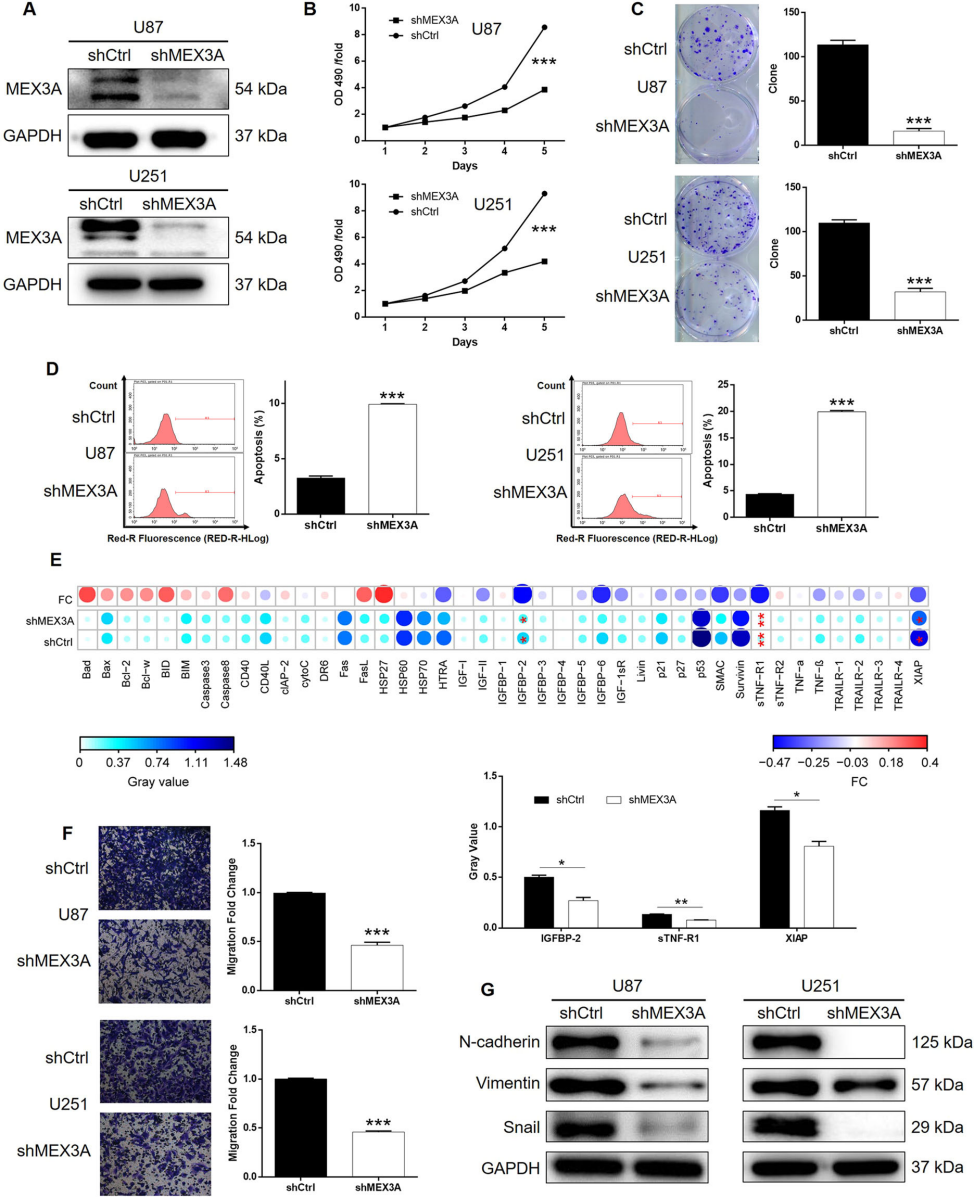
MEX3A knockdown inhibited glioma development in vitro. **A**, **B** Cell models with or without MEX3A knockdown were constructed by transfecting shMEX3A or shCtrl. The knockdown efficiency of MEX3A in BGC-823 and U251 cells was assessed by western blotting (**A**). **B** MTT assay was employed to show the effects of MEX3A on cell proliferation of U87 and U251 cells. **C** The number of colonies formed by U87 and U251 was counted to show the effects of MEX3A on their colony formation ability. **D** Flow cytometry was performed to detect cell apoptosis of U87 and U251 cells with or without MEX3A knockdown. **E** Human apoptosis antibody array was utilized to analyze the regulatory ability of MEX3A on the expression of apoptosis-related proteins in U251 cells. **F** The effects of MEX3A on the cell migration ability of U87 and U251 cells were evaluated by Transwell assay. **G** The expression of EMT-related proteins, including N-cadherin, Vimentin, and Snail, was detected by western blotting in U87 and U251 cells of shMEX3A and shCtrl groups. The representative images were selected from at least three independent experiments. Data were shown as mean ± SD. *P < 0.05, **P < 0.01, ***P < 0.001.

For carrying out loss-of-function experiments, MEX3A-knockdown glioma cell models and the corresponding negative control were constructed through lentivirus-mediated transportation of shMEX3A and shCtrl based on U87 and U251 cells. The validity of cell model construction was verified by the >80% transfection efficiency of lentivirus and the significantly downregulated MEX3A expression in shMEX3A cells (Figs. S1 and 2A). A series of cell phenotypes was subsequently evaluated using the glioma cells with or without MEX3A knockdown. The results of MTT and colony formation assays showed that cell proliferation was significantly decreased in shMEX3A cells compared with the corresponding shCtrl cells (Fig. 2B, C). Conversely, cell apoptosis was significantly promoted in glioma cells with MEX3A knockdown (Fig. 2D). Subsequently, an antibody array was performed to expose the regulatory effects of MEX3A knockdown on the expression of apoptosis-related proteins, which indicated the significant downregulation of IGFBP-2, sTNF-R1, and XIAP (*P* < 0.05, Figs. S2 and 2E). Considering the high aggressiveness of glioma, the regulation of MEX3A on cell migration was also assessed by Transwell assay, showing the observably suppressed migration ability by MEX3A knockdown (Fig. 2F). Furthermore, the effects of MEX3A on glioma cell migration could be partially explained by the downregulated expression of EMT-related proteins, including N-cadherin, Snail, and Vimentin (Fig. 2G).

### MEX3A knockdown inhibited tumor growth of glioma in vivo

**Fig. 3:**
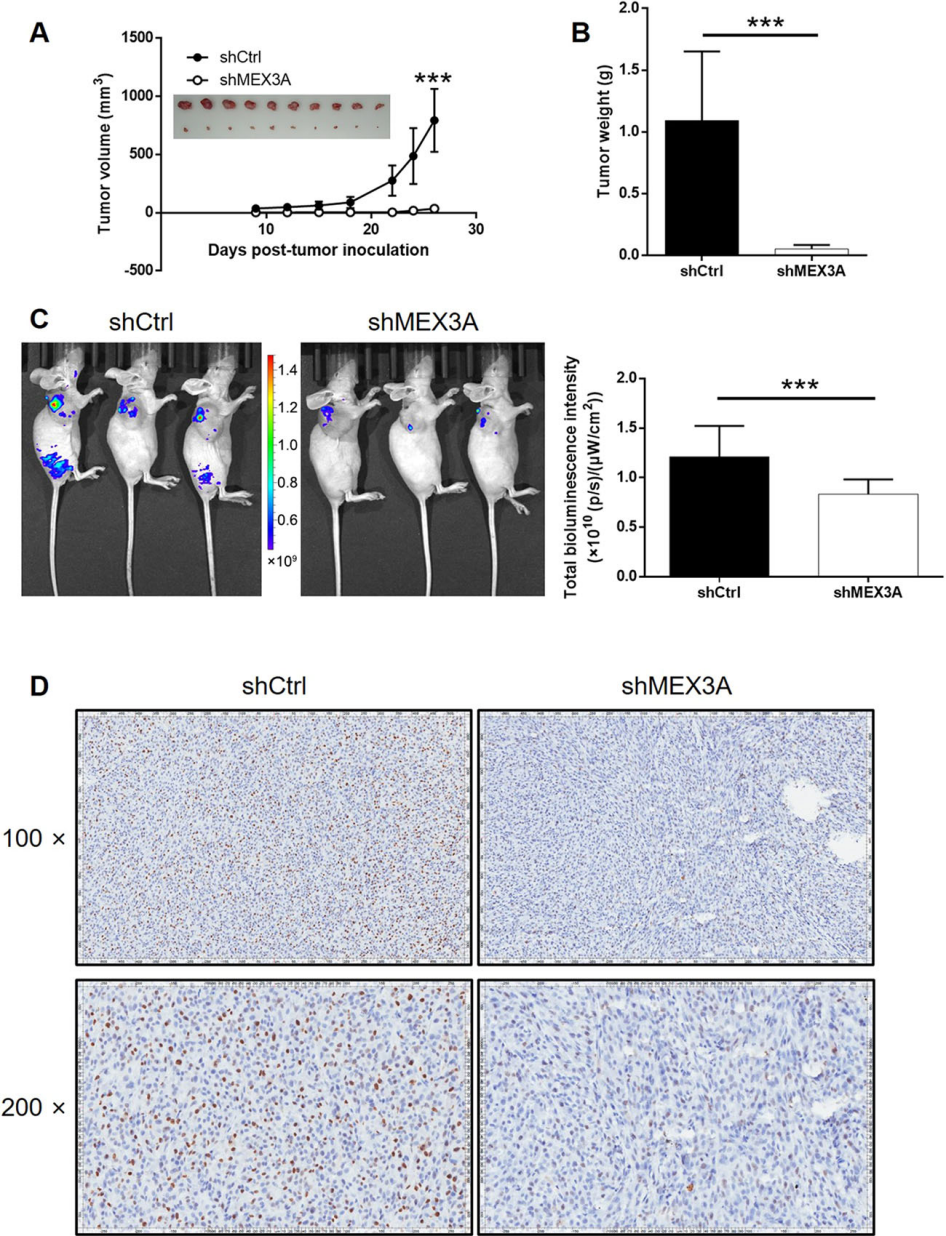
MEX3A knockdown inhibited glioma development in vivo. **A** Seven days post injection of U251 cells with or without MEX3A knockdown, the volume of tumors formed in mice was measured and calculated at the indicated time intervals. Inset: tumors were removed 26 days post injection for collecting photos. B Mice were sacrificed at day 26 post injection, and the tumors were removed for weighing. **C** In vivo imaging was performed to evaluate the tumor burden in mice of shMEX3A and shCtrl groups at day 26 post tumor inoculation. The bioluminescence intensity was scanned and used as a representation of tumor burden in mice of shMEX3A and shCtrl groups. **D** The expression of Ki-67 was detected by IHC to show the proliferative activity of tumors. Data were shown as mean ± SD. *P < 0.05, **P < 0.01, ***P < 0.001.

To further estimate the functions of MEX3A in glioma development in vivo, the tumorigenicity of shMEX3A and shCtrl U87 cells was evaluated after being injected into nude mice. As concluded from the observation of removed tumors, the measurement of tumor volume and weight, and the detection of in vivo fluorescence, xenografts formed by shMEX3A cells grew slower and were smaller and lighter than the ones formed by shCtrl cells (Fig. 3A–C). In addition, the detection of Ki-67 in sections of xenografts was performed to determine whether the inhibition of tumor growth by MEX3A was attributable to proliferation, which showed significantly lower expression of Ki-67 in shMEX3A xenografts (Fig. 3D). All the in vivo experiments give results that agree with the in vitro studies, showing the inhibition of glioma by MEX3A knockdown.

### The potential of CCL2 as the downstream of MEX3A in the regulation of glioma

**Fig. 4:**
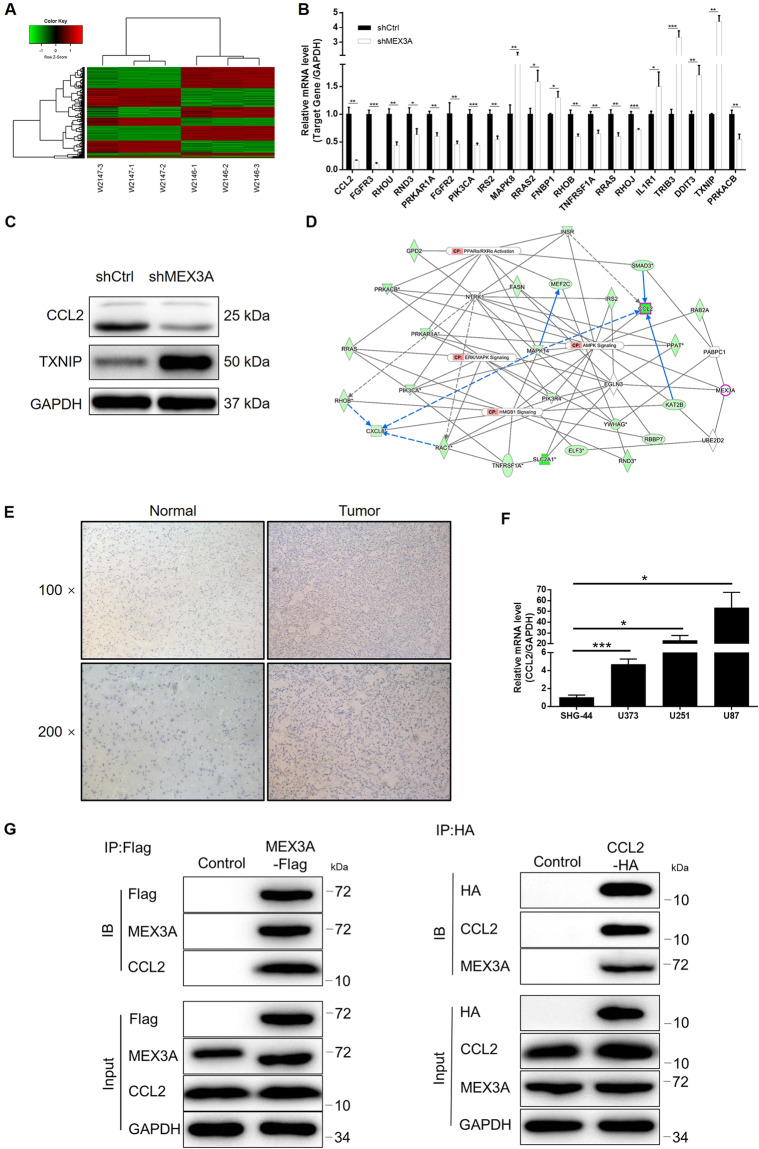
The exploration and verification of downstream-underlying MEX3A-induced regulation of glioma. **A** A PrimeView human gene expression array was performed to identify the differentially expressed genes (DEGs) between shMEX3A and shCtrl groups of U251 cells. **B** qPCR and **C** western blotting were used to detect the expression of several selected DEGs in U251 cells with or without MEX3A. **D** A MEX3A-associated interaction network constructed by IPA analysis revealed the potential linkage between MEX3A and CCL2. **E** The expression of CCL2 in glioma and normal tissues was evaluated by IHC analysis. **F** The mRNA expression of MEX3A in U87, U251, U373, and SHG-44 cell lines was detected by qPCR. **G** A co-IP assay was performed to show the direct interaction between MEX3A and CCL2. The representative images were selected from at least three independent experiments. Data were shown as mean ± SD. *P < 0.05, **P < 0.01, ***P < 0.001.

Next, as the mechanistic study, a PrimeView Human Gene Expression Array was used to show the alteration of gene expression profiling brought by MEX3A knockdown. The comparison between transcription expression profiles of shMEX3A and shCtrl U87 cells identified 687 upregulated differentially expressed genes (DEGs) and 855 downregulated DEGs (Figs. 4A, S3A, B). Subsequently, the bioinformatics analysis based on the IPA database revealed that the most enriched canonical signaling pathway is “PPARα/RXRα activation” and “AMPK signaling” (Fig. S3C), while the most enriched IPA disease and function is “cancer” (Fig. S3D). Based on the combination of the above analysis, expression of several selected DEGs in shMEX3A and shCtrl cells was detected by quantitative polymerase chain reaction (qPCR), among which CCL2 and TXNIP were further subjected to protein-level detection by western blotting (Fig. 4B, C). Subsequently, following the construction of a molecular interaction network revealing the potential linkage between MEX3A and CCL2 (Fig. 4D), we deduced that CCL2 may be a downstream target of MEX3A in the regulation of glioma. As expected, the IHC analysis showed that CCL2 was also upregulated in glioma tissues compared with normal tissues (Fig. 4E); the qPCR measurement showed the abundant expression of CCL2 in U87, U251, U373, and SHG-44 cells (Fig. 4F). More importantly, a co-IP assay clearly demonstrated the direct interaction between MEX3A and CCL2 (Fig. 4G). Collectively, all the above results identified that MEX3A may promote glioma development through regulating CCL2.

### Knockdown of CCL2 blocked development of glioma in vitro

**Fig. 5:**
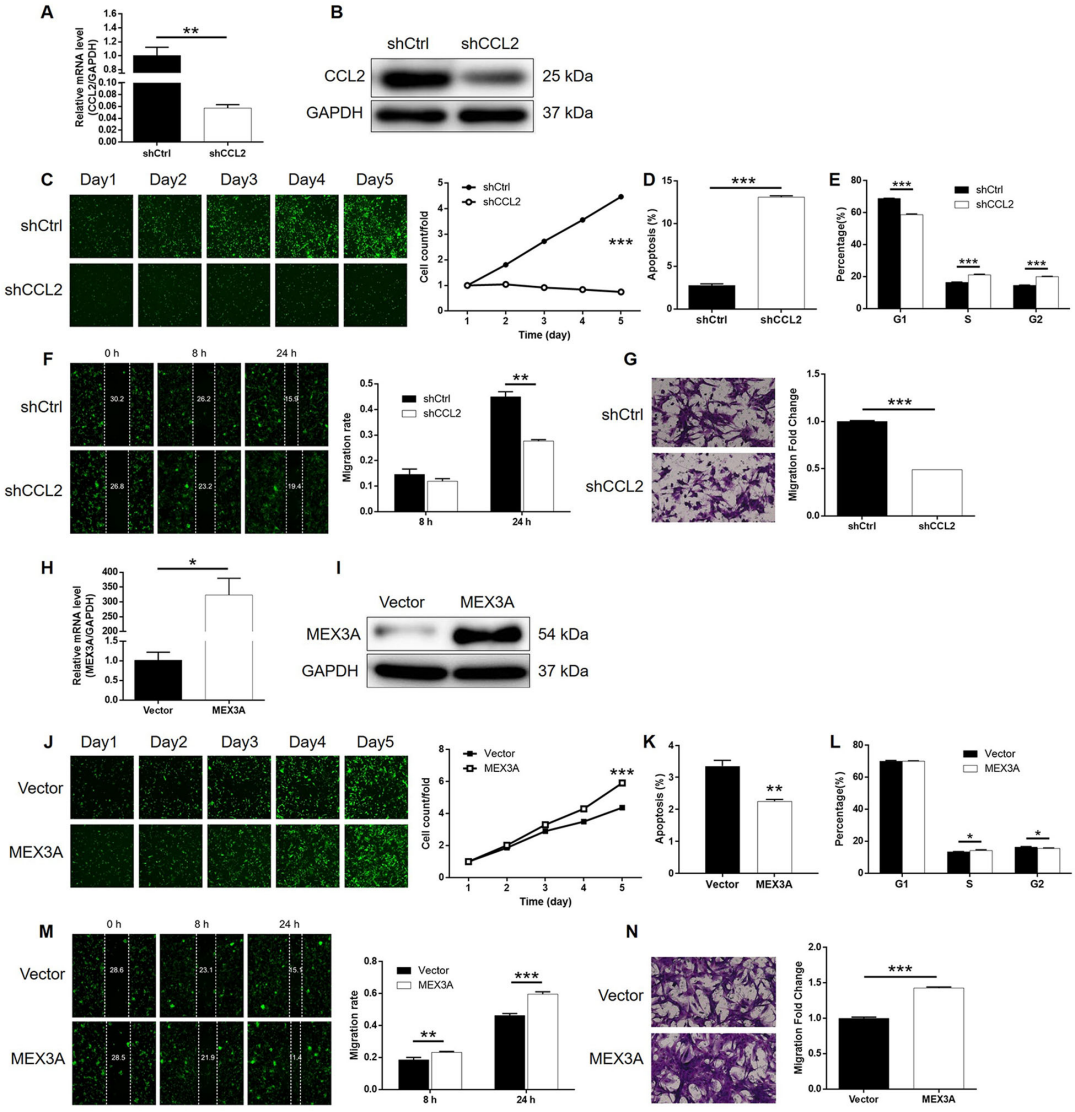
CCL2 knockdown inhibited glioma development, MEX3A overexpression promoted glioma development in vitro. **A**, **B** Cell models with or without CCL2 knockdown were constructed by transfecting shCCL2 or shCtrl. The knockdown efficiency of CCL2 in U251 cells was assessed by qPCR (**A**) and western blotting (**B**). **C** Celigo cell counting assay was employed to show the effects of CCL2 on cell proliferation of U251 cells. Flow cytometry was performed to detect cell apoptosis (**D**) and cell cycle distribution (**E**) of U251 cells with or without CCL2 knockdown. **F**, **G** The effects of CCL2 on the cell migration ability of U251 cells were evaluated by wound-healing assay (**F**) and Transwell assay (**G**). **H**, **I** Cell models with or without MEX3A overexpression were constructed by transfecting control or MEX3A-overexpression plasmids. The overexpression efficiency of MEX3A in U251 cells was assessed by qPCR (**H**) and western blotting (**I**). **J** Celigo cell counting assay was employed to show the effects of MEX3A on cell proliferation of U251 cells. Flow cytometry was performed to detect cell apoptosis (**K**) and cell cycle distribution (**L**) of U251 cells with or without MEX3A overexpression. **M**, **N** The effects of MEX3A on the cell migration ability of U251 cells were evaluated by wound-healing assay (**M**) and Transwell assay (**N**). The representative images were selected from at least three independent experiments. Data were shown as mean ± SD. *P < 0.05, **P < 0.01, ***P < 0.001.

Next, we implemented a set of cell phenotype tests that are similar to MEX3A-related studies on SHG-44 cells transfected with shCCL2 or shCtrl to verify the role of CCL2 in glioma. As mentioned above, the validity of transfection and CCL2 knockdown was inspected by fluorescence imaging, qPCR, and western blotting, respectively (Figs. S5, 5A, and 5B). Similar inhibitory effects of CCL2 knockdown on glioma cell growth were observed by Celigo cell counting assay (Fig. 5C). Meanwhile, the significantly enhanced cell apoptosis and larger cell population arrested in the G2 phase of the cell cycle were revealed by flow cytometry (Fig. 5D, E). Furthermore, both wound-healing and Transwell assays pointed out the significantly suppressed cell migration ability of SHG-44 cells with CCL2 knockdown (Fig. 5F, G). Altogether, the results demonstrated that CCL2 not only has a co-expression profile with MEX3A but also possesses similar regulatory effects on glioma with MEX3A.

### CCL2 knockdown alleviated MEX3A overexpression-induced promotion of glioma

Finally, SHG-44 cells with overexpressed MEX3A (MEX3A group) and with both overexpressed MEX3A and downregulated CCL2 (MEX3A + shCCL2 group) were constructed along with their corresponding negative control and verified (Figs. S5 and 5H–J). Not surprisingly, MEX3A overexpression induced totally adverse effects against MEX3A or CCL2 knockdown on glioma development, including significantly increased cell proliferation rate (Fig. 5J), inhibited cell apoptosis (Fig. 5K), slightly decreased cell cycle arrest of G2 phase (Fig. 5L), and significantly suppressed cell migration capability (Fig. 5M, N). More importantly, the analysis of the results obtained from the MEX3A and MEX3A + shCCL2 group demonstrated that all the effects caused by MEX3A overexpression could be alleviated or even reversed by CCL2 knockdown (*P* < 0.001, Figs. S5 and 6A–F). In other words, these results clarified that MEX3A could promote the development of glioma, in which CCL2 may be involved.

## Discussion

Glioma, a heterogeneous tumor with a high incidence and death rate, is one of the most aggressive malignant tumors diagnosed in the central nervous system^6^. Till now, glioma has been considered as a major threat to human health based on its specific characteristics, including the high proliferative and invasive ability and the transformation tendency of low-grade toward high-grade gliomas^21^. However, due to lack of specific radiotherapy or chemotherapy, patients with glioma are often prone to relapse, the overall treatment efficiency and prognosis are poor, and the survival rate remains very low^22^. Although the pathogenesis and the molecular mechanism of glioma are still unclear, accumulating evidence showed that in the development of glioma, the degree of glioma deterioration could be controlled or even reversed in a certain way such as the abnormal expression or mutation of genes. For example, the results from Azambuja et al.^23^ pointed out that the abilities of glioma cell migration, invasion, and proliferation were reduced by CD73 downregulation followed by the decrease in metalloproteinase-2 and Vimentin expression. Moreover, an investigation concerning the association between CD73 and glioblastoma revealed that CD73 inhibition made the progression of rat glioblastoma slower in vivo. Another report by Fernandez-Luna et al. mentioned that a good agreement was reached between ODZ1 and maintenance of glioblastoma aggressiveness via a Myc-dependent transcriptional upregulation of RhoA^24^. Benefitting from the recognition of glioma biomarkers such as O^6^-methylguananine–DNA methyltransferase (MGMT) and epidermal growth factor receptor, patients with glioma have been diagnosed as early as possible, but the overall 5-year survival rate is still far from satisfaction^25,26^.

MEX3A, as one of the MEX3 homologous genes, is an important part of cytoplasmic processing. Given the fact that MEX3A participates in the degradation or inhibition of mRNA by ubiquitination, a lot of studies provided evidence that MEX3A played an important role in the occurrence and development of many diseases including cancer. Regarding the current study, the generally higher MEX3A expression was detected in glioma tissues compared with that in normal tissues, which was also verified by the data mining of TCGA. In addition, patients with relatively higher MEX3A expression were often diagnosed as advanced with a shorter survival period, which implied the potential role of MEX3A in the development of glioma. Subsequently, the potential role of MEX3A in glioma was validated by the in vitro experiments in which MEX3A knockdown inhibited glioma cell proliferation and motility, and promoted glioma cell apoptosis. Furthermore, the alteration of apoptosis- or EMT-related proteins caused by MEX3A knockdown made clear the regulatory mechanism of cell apoptosis and migration. In terms of the results of the observation and measurement of tumor-bearing mice models, the promotor role of MEX3A in the development and progression of glioma was further clarified in vivo. In previous studies, Jiang et al.^18^ revealed that MEX3A was involved in gastric cancer cell transformation based on the results that MEX3A knockdown effectively inhibited the proliferation of gastric cancer cells and colony formation. At the same time, the migration of cells was significantly inhibited by the knockdown of MEX3A in gastric cancer cells. On the other hand, MEX3A expression was significantly upregulated in gastric cancer tissues relative to normal tissues, indicating MEX3A as a participant in development and progression of gastric cancer. Moreover, it was reported by Pereira et al. that MEX3A participated in the regulation of CDX2 in colorectal cancer and damaged the differentiation of intestinal cells, suggesting that MEX3A may be an oncogene for colorectal cancer^19^. Considerable research efforts showed that the downregulation of MEX3A in bladder cancer could inhibit the proliferation of cells, promote cell apoptosis, thereby exerting a therapeutic effect to inhibit the further deterioration of bladder cancer cells^27,28^. In addition, Krepischi et al.^20^ found that MEX3A was overexpressed in nephroblastoma by microarray comparative genomic hybridization (Array-CGH). To the best of our knowledge, this is the first to report the oncogene-like functions of MEX3A in glioma.

**Fig. 6:**
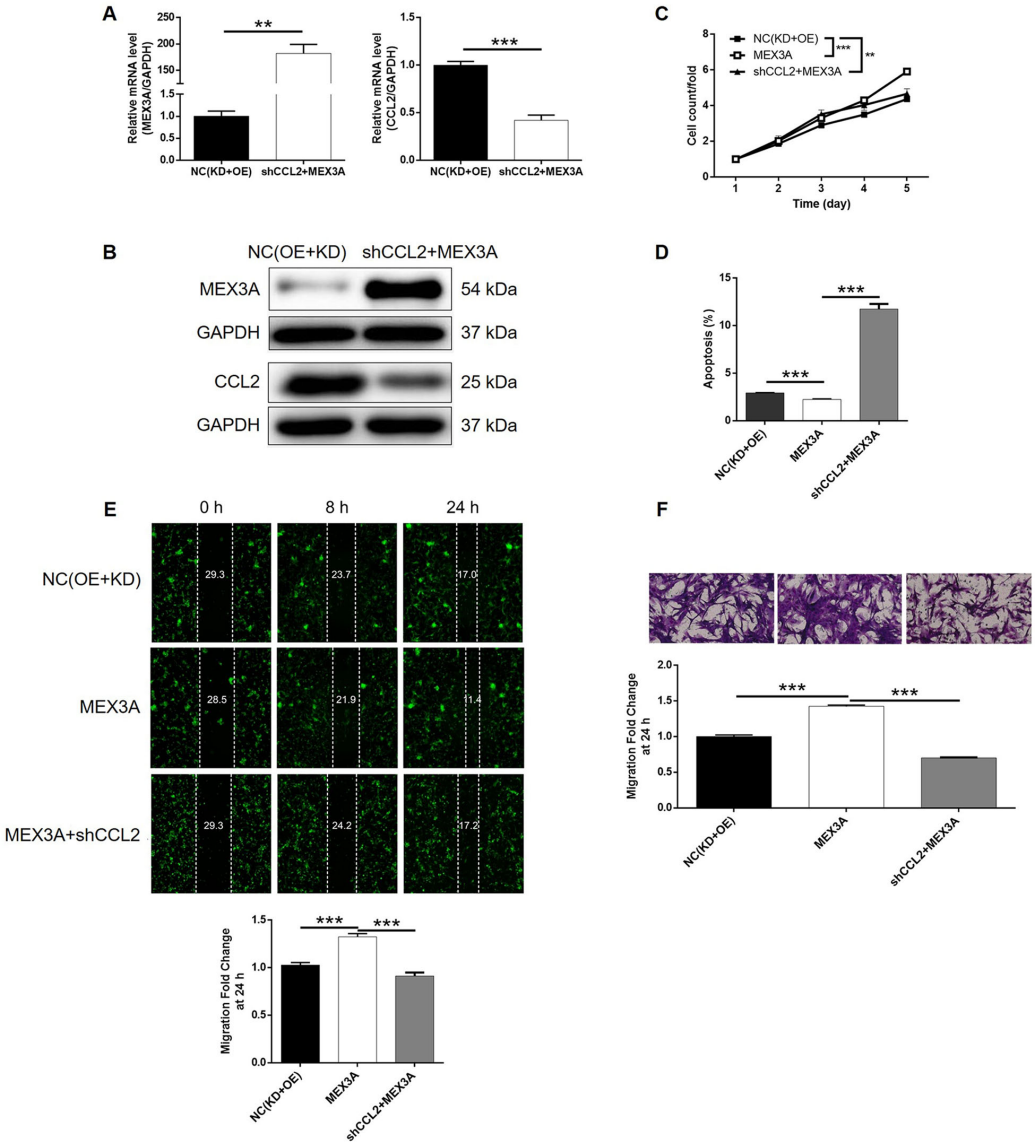
Knockdown of CCL2 attenuated the effects of glioma cells by MEX3A overexpression. U251 cells transfected with NC(OE + KD), MEX3A-overexpression plasmids, and simultaneous MEX3A-overexpression plasmids, and shCCL2 was subjected to the detection of expression (**A**, **B**) cell proliferation by Celigo cell counting assay (**C**), cell apoptosis by flow cytometry (**D**), cell migration by wound-healing assay (**E**), and cell migration by Transwell assay (**F**). The representative images were selected from at least three independent experiments. Data were shown as mean ± SD. *P < 0.05, **P < 0.01, ***P < 0.001.

The mechanistic research of this study revealed CCL2 as a potential downstream target of MEX3A in the regulation of glioma (Fig. 6). Herein, we verified that, similar with MEX3A, CCL2 was also upregulated in glioma tissues relative to normal tissues. The following phenotype detection comparing proliferation, apoptosis, and migration of glioma cells with or without CCL2 knockdown further verifies the inhibitory role of CCL2 in glioma. More importantly, we further illustrated that knockdown of CCL2 could alleviate or reverse the promotion effects of MEX3A overexpression on glioma, indicating its potential role as the downstream of MEX3A. Actually, in previous work published by Li et al.^29^, CCL2 was also found to be downregulated by knockdown of HOXC10, which promotes proliferation and invasion and induces immunosuppressive gene expression in glioma. Tumor microenvironment, which refers to the internal and external environment of tumor cells, is the key factor of tumor growth, diffusion, and metastasis^30,31^. Monocyte chemoattractant protein-1 (MCP-1, alias CCL2), as an important chemokine in the tumor microenvironment, is one of the important members of CC subfamily^32,33^. More importantly, CCL2 can be produced not only by a variety of activated cells, such as fibroblasts, lymphocytes, and macrophages in tumor microenvironment, but also by a variety of tumor cells^34,35^. CCL2 can bind to its receptor CCR2 and play an important role in tumor development and metastasis by regulating tumor cell growth and survival, angiogenesis, tumor invasion, and metastasis^36,37^. Recently, Chen et al.^38^ released that CCL2 could act as a key target of ELF-1 contributing to the cell proliferation, migration, invasion, and drug resistance of nasopharyngeal carcinoma. Meanwhile, CCL2 was also reported to be a potential downstream target of Notch1, which is uncovered as a novel oncogenic protein in nasopharyngeal carcinoma^39^. The study of Zhou et al.^40^ showed that activation of the neddylation pathway is capable of promoting tumor-associated macrophage infiltration through facilitating CCL2 transactivation via NF-κB-CCL2 signaling in lung cancer. Besides, the biological functions of CCL2 in glioma have also been comprehensively investigated and have been understood to some extent^41–44^.

Conclusively, our study demonstrated the role played by MEX3A in glioma that MEX3A was a glioma tumor promotor. The upregulated or downregulated MEX3A expression in glioma regulated the development and progression of glioma through targeting CCL2, which provided a reference that MEX3A may be served as a prognostic marker and a novel therapeutic target in glioma treatment.

## Materials and methods

### Cells and clinical tissue microarray

U87, U251, U373, and SHG-44 cell lines were purchased from BeNa Technology (China). U87, SHG-44, and U251 were cultured in 90% DMEM-H with 10% fetal bovine serum (FBS) and U373 was cultured in 90% RPMI-1640 with 10% FBS.

Formalin-fixed glioma tissues and normal tissue chip were obtained from Shanghai Outdo Biotech Company (# HBraG180Su01). Tissues were collected from 2009.2 to 2017.7. All patients were completely informed before the collection of the tissue samples and written informed consent was provided as well.

### IHC staining

After being dewaxed, rehydrated, and blocked, slides were incubated with MEX3A antibody (1:100, Abcam) at 4 °C overnight, then washed with phosphate-buffered saline (PBS) 3 times, and incubated with horseradish peroxidase (HRP)-conjugated goat anti-rabbit IgG polyclonal antibody for 30 min at room temperature. DAB and hematoxylin were used to stain tissue slides. IHC scoring of specimens was determined based on the sum of the staining intensity and its extent scores. Antibodies used in IHC staining were listed in Table S1.

### Target gene knockdown cell models and cell transfection

The target sequences of MEX3A and CCL2 RNA interference and the amplification sequence of primers for MEX3A were designed in Shanghai Biosciences, Co., Ltd. A single-strand DNA oligo containing interference sequence was synthesized and annealed for pairing to produce double-strand DNA. Then the two ends of the oligo were connected to the BR-V-108 lentivirus vector and transferred into the top ten *E. coli* receptor cells (TIANGEN). PCR was used to identify the positive recombinants, which were then DNA-sequenced for verification. Plasmid extraction was carried out for the correct clones using EndoFree Maxi Plasmid Kit (TIANGEN, #DP117). The information of sequences could be found in Table S2.

U87 and U251 cells were cultured in DMEM with 10% FBS and infected with 1 × 10^7^ TU/mL lentivirus vectors with ENI.S and Polybrene at a Mol of 5 by Lipofectamine 2000 (Invitrogen). The fluorescence of cells and infection efficiency was observed by fluorescence microscope (OLYMPUS).

### Real-time quantitative PCR

Total RNA from SHG-44, U87, U251, and U373 cells was isolated, respectively, using TRIZOL (Sigma) following the manufacturer’s instructions. Reverse transcription of RNA (2.0 μg) to cDNA was performed using Hiscript QRT supermix for qPCR (+gDNA WIPER) (Vazyme) following the manufacturer’s instructions. Real-time qPCR was carried out using AceQ qPCR SYBR Green master mix (Vazyme) on the VII7 real-time PCR instrument. The 2^−ΔΔCt^ method was used to analyze the relative quantitation of target genes with GAPDH as the internal reference. Primers for genes were synthesized by Genewiz and shown in Table S3.

### Western blot

Cells were lysed in ice-cold radioimmunoprecipitation assay buffer (Millipore) and protein concentration was detected by BCA Protein Assay Kit (HyClone-Pierce). The same amount of total protein from each group was separated by 10% sodium dodecyl sulfate–polyacrylamide gel electrophoresis and electrotransferred onto polyvinylidene difluoride membranes (Bio-Rad Laboratory). The membranes were blocked in TBST plus 5% nonfat milk, and incubated with primary antibodies. After washing with TBST, membranes were incubated with the appropriate secondary antibody. The signals were visualized using ECL-PLUS Kit (Amersham). Band intensities were quantified using ImageJ software (NIH). Antibodies were detailed in Table S1.

### MTT assay

Lentivirus-infected U87 and U251 (LV-shMEX3A and LV-Ctrl) cells were measured by MTT assay. Cells in the exponential growth phase were seeded onto 96-well plates (2,000 cells/well). MTT solution (20 μL, 5 mg/mL, Gongsheng) was incubated with cells for 4 h. The OD_490_ was measured by a microplate reader (Tecan) at days 1–5, which reflects the number of viable cells. The cell viability ratio was calculated.

### Celigo cell counting assay

Seventy-two hours after the infection, SHG-44 cells were seeded into a 96-well plate with a cell density of 2000 cells/well in DMEM medium containing 10% FBS and further cultured for 5 days. Cell counting was accomplished every day by Celigo image cytometer (Nexcelom Bioscience) and the cell proliferation curve was drawn.

### Cell apoptosis

LV-shMEX3A and LV-Ctrl-infected cells were inoculated in a 6-well plate and cells were harvested when the cell density reached 85%. After washing with 4 °C ice-cold PBS, cells were centrifuged at 1200 rmp and resuspended with 100 μL of binding buffer. Evaluation of apoptosis was performed by Annexin V-APC staining flow cytometry method (eBioscience) according to the manufacturer’s protocol.

### Cell cycle

Infected SHG-44 cells were seeded in a 6-cm dish and cultured for about 5 days. Cells were harvested and centrifuged at 1200 rmp, washed with 4 °C ice-cold PBS, and cells were fixed by 75% 4 °C ice-cold alcohol. Then cells were stained by Annexin V-APC and PI reagents for 30 min at room temperature in the dark. The percentage of cells in G1, S, and G2–M phases was analyzed.

### Colony formation assay

Exponentially growing lentivirus-infected U87 and U251 (LV-shMEX3A and LV-Ctrl) cells were inoculated in a 6-well plate with 600 cells/well. Cell clones were photographed by an Olympus digital camera, and then fixed and stained by 4% paraformaldehyde and GIEMSA (DingGuo Biotechnology), respectively. The fluorescence photos of clones were collected and numbers of the colony were counted.

### Transwell migration assay

Migration assay was performed using Corning transwell chambers. Exponentially growing lentivirus-infected U87, U251, and SHG-44 cells were seeded in a 24-well plate with 1 × 10^5^ cells/well in which the upper chambers were filled with 100 μL of serum-free medium. About 600 μL of complete medium was filled in the lower chamber and incubated at 37 °C for 16 h. At the end of incubation, floating cells were removed and cells in the lower chamber were fixed and stained with Giesma. Images of cells were taken and analyzed using NIH imageJ software.

### Wound-healing assay

Lentivirus-infected SHG-44 cells (4 × 10^4^ cells/well) were plated into a 96-well plate in triplicate for culturing. After the confluence of cells reached 90%, the medium was exchanged to medium with 0.5% FBS. Scratches were created with a 96-wounding replicator (VP scientific) and the cell debris was washed with PBS. Photographs were taken by a fluorescence microscope at 8 and 24 h post scratching and cell migration rate was calculated.

### Protein array analysis

Analysis of total proteins collected from shCtrl and shMEX3A U251 was conducted using Human Apoptosis Array according to the manufacturer’s instructions. First, the array was incubated with protein sample at 4 °C overnight. After washing three times, the array was incubated with the primary antibodies for 2 h at room temperature and further incubated with the secondary antibody for 1 h. Protein spots of the array were visualized by chemiluminescence detection reagents, and the intensity of the spot was measured with the ImageJ software.

### GeneChip

There was detection of gene expression profile in U251 cells infected with shCtrl or shMEX3A by RNA screening analysis using GeneChip prime view human assay kit (PathArray^TM^) following the manufacturer’s instructions. Total RNA was extracted by the RNeasy kit (Sigma). Concentration and values of A260 and A280 of total RNA were determined by Nanodrop 2000 (Thermo Fisher Scientific). RIN value was evaluated with Agilent 2100 and Agilent RNA 6000 Nano Kit (Agilent). RNA sequencing was performed with Affymetrix human GeneChip PrimeView according to the manufacturer’s instruction and the outcomes were scanned by Affymetrix Scanner 3000 (Affymetrix). Raw data statistical significance assessment was accomplished using a Welch *t* test with Benjamini–Hochberg FDR. Significant difference and functional analyses based on IPA (Qiagen) were executed.

### Xenograft model

All animal procedures were approved by Tongji Hospital, Tongji Medical College, Huazhong University of Science and Technology Institutional Review Board of Experimental Animals. Xenograft model in female BALB/c nude mice was formulated by subcutaneous injection of 0.2 mL of exponentially growing lentivirus-infected U87 cell suspensions at a density of 2 × 10^7^ cell/mL. Mice were purchased from Shanghai Lingchang Experimental Animals Co., Ltd. Tumor growth was assessed twice a week using a caliper and tumor volumes (*V*) were estimated as *V* = *π*/6 × *L* × *W* × *W* (*L* and *W* were tumor length and width, respectively). In addition, bioluminescent imaging was applied by giving 10 μL/g d-Luciferin (15 mg/mL, Shanghai Qianchen) via intraperitoneal injection and performed under a Living Image System (Perkin Elmer). After bioluminescent imaging, mice were sacrificed and the tumor tissues were removed for Ki-67 immunostaining.

### Ki-67 staining assay

Tissue slides were blocked with 3% PBS–H_2_O_2_ and were incubated with primary antibody Ki-67 at 4 °C overnight. Then slides were incubated with HRP goat anti-rabbit IgG at room temperature for 2 h. Finally, all slides were stained by Hematoxylin (# BA4041, Baso) and Eosin (# BA4022, Baso).

### Statistical analysis

Cell experiments were performed in triplicate and data were expressed as the mean ± SD, and Student’s *t* test was used to analyze the statistical significance. Multiple groups were compared by one-way ANOVA. All statistical analysis was performed using SPSS 17.0 (IBM, SPSS) and GraphPad Prism 6.01 (GraphPad Software). *P* < 0.05 was considered statistically significant. Statistical methods, including Rank Sum test analysis, two-way ANOVA Mann–Whitney *U* analysis, and Spearman Rank correlation analysis, were applied for statistical analysis. Overall survival analysis was performed using the Kaplan–Meier method.

## Supplementary information

Table S1

Table S2

Table S3

Table S4

Figure S1

Figure S2

Figure S3

Figure S4

Figure S5

Supplementary figure legends
