# Supplementary material for: MEX3A contributes to development and progression of glioma through regulating cell proliferation and cell migration and targeting CCL2
Source: Cell Death Dis. 2021 Jan 4;12(1):14. doi: 10.1038/s41419-020-03307-x (PMC7791131; doi:10.1038/s41419-020-03307-x)
Supplement: Supplementary file 1 — Table S1 [file 41419_2020_3307_MOESM1_ESM.docx]

Table S1 Antibodies used in western blotting and IHC

| Primary antibodies | Dilution in WB | Source species | Company | Catalog No. |
| --- | --- | --- | --- | --- |
| MEX3A | 1:1000 | Rabbit | abcam | ab79046 |
| GAPDH | 1:3000 | Rabbit | Bioworld | AP0063 |
| N-cadherin | 1:1000 | Rabbit | abcam | ab18203 |
| Vimentin | 1:1000 | Rabbit | abcam | ab92547 |
| Snail | 1:1000 | Rabbit | CST | 3879S |
| CCL2 | 1:1000 | Rabbit | abcam | ab25124 |
| TXNIP | 1:1000 | Rabbit | abcam | ab188865 |
| Primary antibodies | Dilution in IHC | Source species | Company | Catalog No. |
| Ki67 | 1:200 | Rabbit | abcam | ab16667 |
| MEX3A | 1:400 | Rabbit | abcam | ab79046 |
| CCL2 | 1:100 | Rabbit | abcam | ab25124 |
|  |  |  |  |  |
|  |  |  |  |  |
| Secondary antibody | Dilution |  | Company | Catalog No. |
| HRP Goat Anti-Rabbit IgG (WB) | 1:3000 |  | Beyotime | A0208 |
| HRP Goat Anti-Mouse IgG (WB) | 1:3000 |  | Beyotime | A0216 |
| HRP Goat Anti-Rabbit IgG (IHC) | 1:400 |  | abcam | ab6721 |
