# Supplementary material for: MEX3A contributes to development and progression of glioma through regulating cell proliferation and cell migration and targeting CCL2
Source: Cell Death Dis. 2021 Jan 4;12(1):14. doi: 10.1038/s41419-020-03307-x (PMC7791131; doi:10.1038/s41419-020-03307-x)
Supplement: Supplementary file 2 — Table S2 [file 41419_2020_3307_MOESM2_ESM.docx]

Table S2 The target sequences and shRNA sequences

| Gene | No. | Target sequence (5'-3') | shRNA sequences (5'-3') |
| --- | --- | --- | --- |
| MEX3A | Pbr-140-a | AGGCAAGGCTGCAAGATTAAG | ccggAGGCAAGGCTGCAAGATTAAGctcgagGCTTAATCTTGCAGCCTTGCCtttttg |
| MEX3A | Pbr-140-b | AGGCAAGGCTGCAAGATTAAG | aattcaaaaaAGGCAAGGCTGCAAGATTAAGctcgagGCTTAATCTTGCAGCCTTGCC |
| CCL2 | Pbr10890-a | AGTCACCTGCTGTTATAACTT | CCGGAGTCACCTGCTGTTATAACTTCTCGAGAAGTTATAACAGCAGGTGACTTTTTTG |
| CCL2 | Pbr10890-b | AGTCACCTGCTGTTATAACTT | AATTCAAAAAAGTCACCTGCTGTTATAACTTCTCGAGAAGTTATAACAGCAGGTGACT |
| CCL2 | Pbr10891-a | TCCCAAAGAAGCTGTGATCTT | CCGGTCCCAAAGAAGCTGTGATCTTCTCGAGAAGATCACAGCTTCTTTGGGATTTTTG |
| CCL2 | Pbr10891-b | TCCCAAAGAAGCTGTGATCTT | AATTCAAAAATCCCAAAGAAGCTGTGATCTTCTCGAGAAGATCACAGCTTCTTTGGGA |
| CCL2 | Pbr10892-a | AAGCAGAAGTGGGTTCAGGAT | CCGGAAGCAGAAGTGGGTTCAGGATCTCGAGATCCTGAACCCACTTCTGCTTTTTTTG |
| CCL2 | Pbr10892-b | AAGCAGAAGTGGGTTCAGGAT | AATTCAAAAAAAGCAGAAGTGGGTTCAGGATCTCGAGATCCTGAACCCACTTCTGCTT |
