# Supplementary material for: MEX3A contributes to development and progression of glioma through regulating cell proliferation and cell migration and targeting CCL2
Source: Cell Death Dis. 2021 Jan 4;12(1):14. doi: 10.1038/s41419-020-03307-x (PMC7791131; doi:10.1038/s41419-020-03307-x)
Supplement: Supplementary file 3 — Table S3 [file 41419_2020_3307_MOESM3_ESM.docx]

Table S3 Primers used in qPCR

| Gene | Forward primer sequence (5’-3’) | Reverse primer sequence (5’-3’) |
| --- | --- | --- |
| CCL2 | TAGAAGAATCACCAGCAGCAAG | CTTCGGAGTTTGGGTTTGC |
| FGFR3 | ACCCTACGTTACCGTGCTCA | GAAATTGGTGGCTCGACAGA |
| RHOU | CGCCTCCTACATCGAGTGTT | GACTTCTTTGGCTGTTGCTGA |
| RND3 | AAGAATAGAGTTGAGCCTGTGGG | GCATCCGAATCAGGGTAAGAG |
| PRKAR1A | TGCCATGTTTTCGGTCTCC | CTGGTTGCCCATTCATTGTT |
| FGFR2 | GTATGAACTTCCAGAGGACCCA | CCATGACCACTTGCCCAAA |
| PIK3CA | AGCAAATGAGGCGACCAGA | GGGTTCTCCCAATTCAACCAC |
| IRS2 | GGCATTCCAGCCCCTATGTT | GAGAACTGCCACACACTGGT |
| MAPK8 | TGCCACAAAATCCTCTTTCCAGG | AGGTCTGTTTTCAACGTAAGTCCT |
| RRAS2 | GAGGCATCAGCAAAGATTAGGA | GGTTCTGGTGAAGGAGGACATT |
| FNBP1 | TTGTGGACTTGGCACGCTAT | TGTGCTGCTGTGCTTTACGG |
| RHOB | TGTGTGTCTGTTCGACTCCC | TATCAAGCTCCCGCTGGTGA |
| TNFRSF1A | GTTGTGCCTACCCCAGATTG | GGAGGGATAAAAGGCAAAGAC |
| RRAS | TGTCTGACTACGACCCCACT | GTACTGCTCTCTCATGGCCC |
| RHOJ | TTGCTAACGGGCCACTCATT | CTCGGGAGCTATTTGCCTGT |
| IL1R1 | GTGGCTGAAAAGCATAGAGGG | GTCTCATTAGCTGGGCTCACA |
| TRIB3 | AGCGGTTGGAGTTGGATGA | TTGCACGATCTGGAGCAGTAG |
| DDIT3 | GAGCTGGAAGCCTGGTATGA | AGAAGCAGGGTCAAGAGTGGT |
| TXNIP | TGTTCCCGAATTGTGGTCC | TGCGCATGTCCCTGAGATAA |
| PRKACB | TGGATTGGTGGGCATTAGG | GAACTGAAGTGGGATGGGAAT |
| CCL2 | TAGAAGAATCACCAGCAGCAAG | CTTCGGAGTTTGGGTTTGC |
