# Supplementary material for: MEX3A contributes to development and progression of glioma through regulating cell proliferation and cell migration and targeting CCL2
Source: Cell Death Dis. 2021 Jan 4;12(1):14. doi: 10.1038/s41419-020-03307-x (PMC7791131; doi:10.1038/s41419-020-03307-x)
Supplement: Supplementary file 4 — Table S4 [file 41419_2020_3307_MOESM4_ESM.docx]

Table S4 Relationship between MEX3A expression and tumor characteristics in patients with glioma analyzed by Spearman rank correlation analysis

| Tumor characteristics | index |  |
| --- | --- | --- |
| Grade | Pearson correlation | 0.196 |
|  | Significance (two tailed) | 0.027* |
|  | n | 128 |
