# Supplementary figures and images for: MEX3A contributes to development and progression of glioma through regulating cell proliferation and cell migration and targeting CCL2
Source: Cell Death Dis. 2021 Jan 4;12(1):14. doi: 10.1038/s41419-020-03307-x (PMC7791131; doi:10.1038/s41419-020-03307-x)

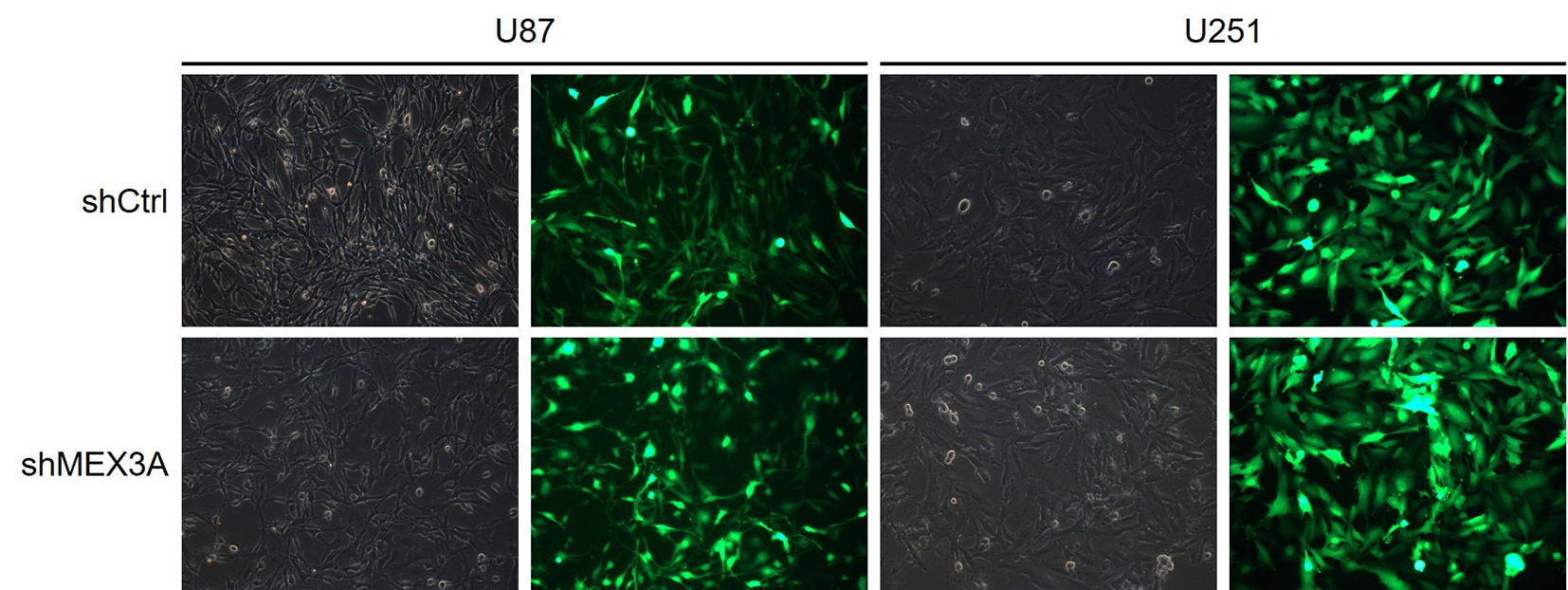

Supplement: Supplementary file 5 — Figure S1 [file 41419_2020_3307_MOESM5_ESM.tif]

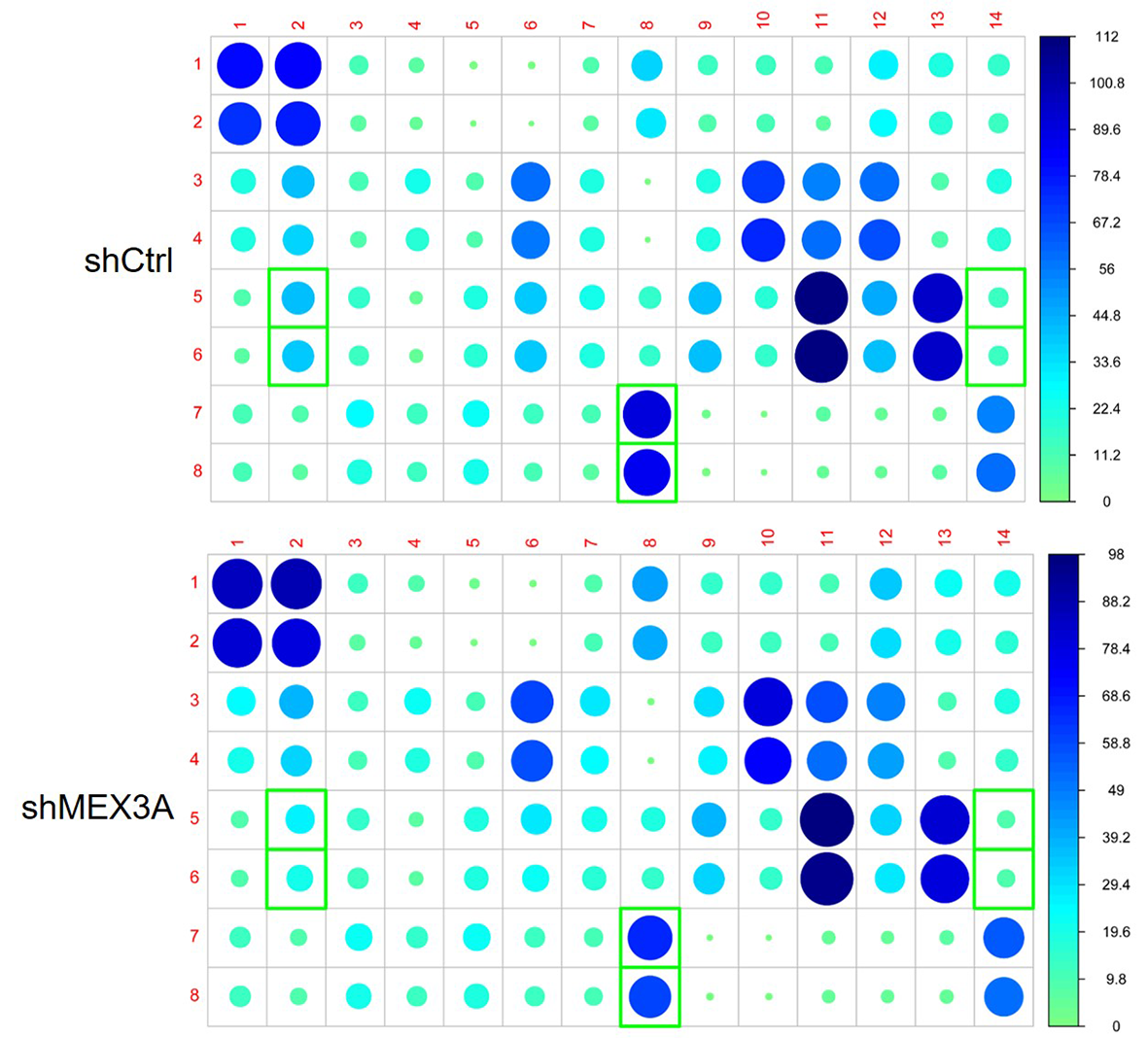

Supplement: Supplementary file 6 — Figure S2 [file 41419_2020_3307_MOESM6_ESM.tif]

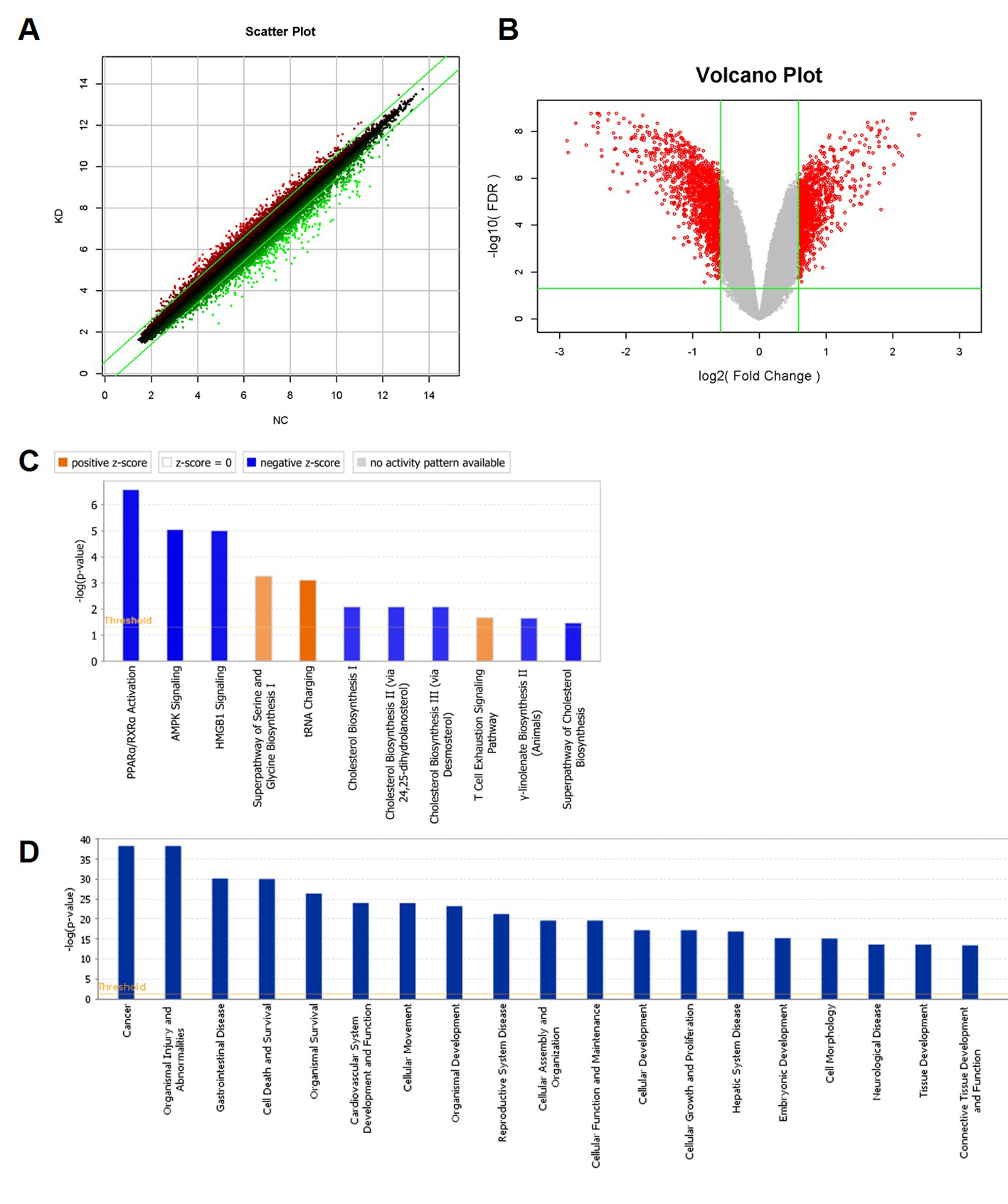

Supplement: Supplementary file 7 — Figure S3 [file 41419_2020_3307_MOESM7_ESM.tif]

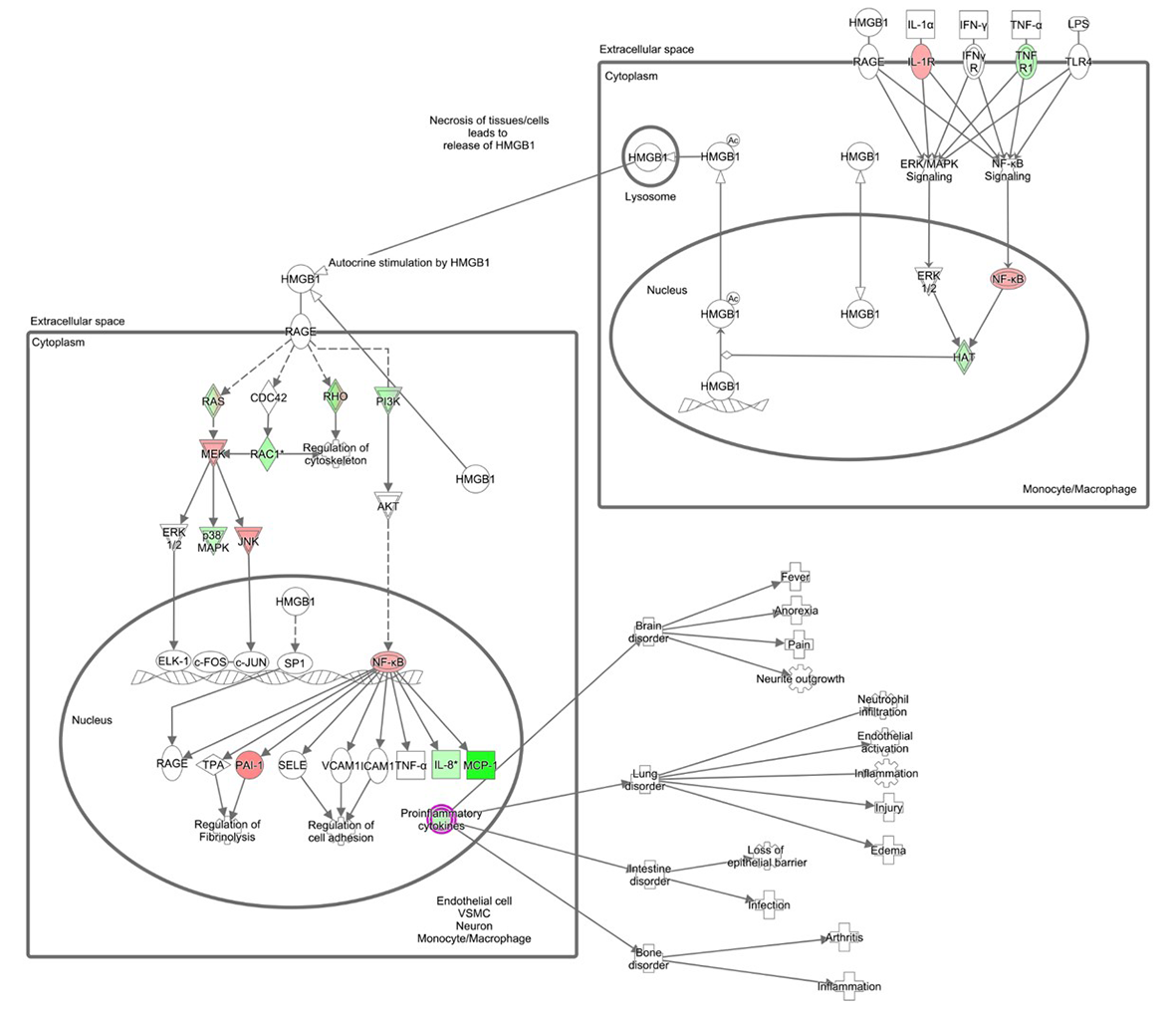

Supplement: Supplementary file 8 — Figure S4 [file 41419_2020_3307_MOESM8_ESM.tif]

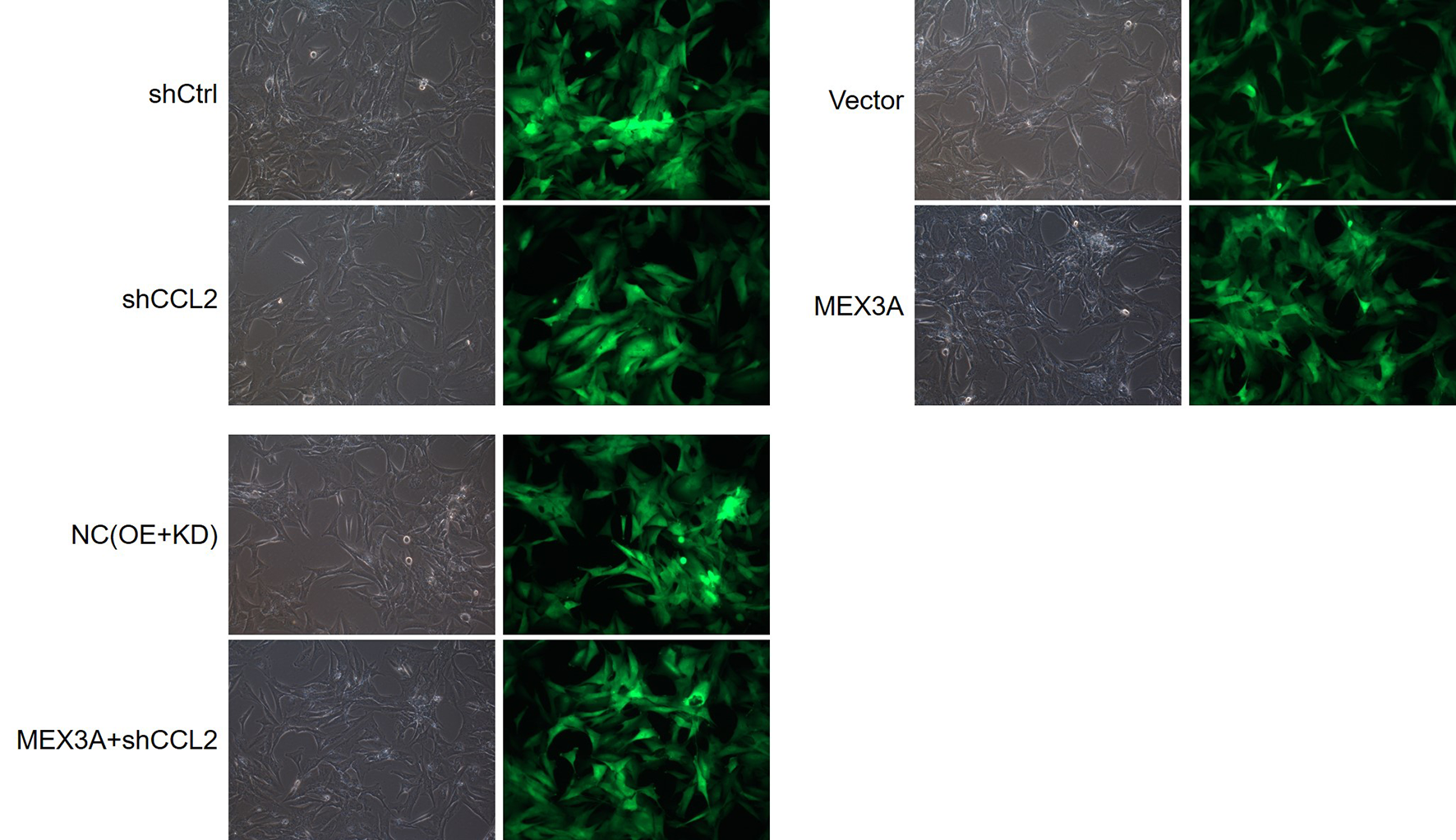

Supplement: Supplementary file 9 — Figure S5 [file 41419_2020_3307_MOESM9_ESM.tif]
