# Supplementary material for: MEX3A contributes to development and progression of glioma through regulating cell proliferation and cell migration and targeting CCL2
Source: Cell Death Dis. 2021 Jan 4;12(1):14. doi: 10.1038/s41419-020-03307-x (PMC7791131; doi:10.1038/s41419-020-03307-x)
Supplement: Supplementary file 10 — Supplementary figure legends [file 41419_2020_3307_MOESM10_ESM.docx]

**Figure S1.** The transfection efficiencies of shMEX3A and shCtrl in U87 and U251 cells were evaluated through observing the fluorescence of GFP on lentivirus vector.

**Figure S2.** Human Apoptosis Antibody Array was performed to detect and compare the expression of apoptosis-related proteins in U251 cells with or without MEX3A knockdown.

**Figure S3.** (A) The scatter plot of gene expression profiling in U251 cells with or without MEX3A knockdown. Red dots represented significantly upregulated DEGs. Green dots represented significantly downregulated DEGs. (B) The volcano plot of gene expression profiling in U251 cells with or without MEX3A knockdown. Red dots represented the DEGs. (C) The enrichment of the DEGs in canonical signaling pathways was analyzed by IPA. (D) The enrichment of the DEGs in IPA disease and function was analyzed by IPA. Data was shown as mean ± SD. **P* < 0.05, ***P* < 0.01, ****P* < 0.001

**Figure S4.** The schematic diagram of bioinformatics analysis identifying CCL2 (MCP-1) as potential target of MEX3A.

**Figure S5.** The transfection efficiencies of shCCL2, shCtrl, Control plasmid (Vector), MEX3A overexpression plasmid, NC(OE+KD) and MEX3A+shCCL2 in U251 cells were evaluated through observing the fluorescence of GFP on lentivirus vector.
